# High-Power LED Units Currently Available for Dental Resin-Based Materials—A Review

**DOI:** 10.3390/polym13132165

**Published:** 2021-06-30

**Authors:** Rita Almeida, Patricia Manarte-Monteiro, Joana Domingues, Carlos Falcão, Mariano Herrero-Climent, Blanca Ríos-Carrasco, Bernardo Ferreira Lemos

**Affiliations:** 1 Faculty of Health Sciences, University Fernando Pessoa, 4200-150 Porto, Portugal; almeidasousanarita@gmail.com; 2 Department of Dentistry and Medical Sciences, Faculty of Health Sciences, University Fernando Pessoa, 4200-150 Porto, Portugal; patmon@ufp.edu.pt (P.M.-M.); joanad@ufp.edu.pt (J.D.); cfalcao@ufp.edu.pt (C.F.); blemos@ufp.edu.pt (B.F.L.); 3 Porto Dental Institute, 4150-518 Porto, Portugal; dr.herrero@herrerocliment.com; 4 Department of Periodontology, University of Seville, 41009 Seville, Spain

**Keywords:** dental curing units, LED curing-lights, composite resins, adverse events, dental materials

## Abstract

The pursuit of less time-consuming procedures led to the development of high-power light-curing-units (LCU) to light-cure dental-resin-based-materials. This review aims to describe high-power light-emitting-diode (LED)-LCUs, by a bibliometric systematization of in vitro and in vivo studies. The research-question, by PICO model, aimed to assess the current knowledge on dentistry-based high-power LED-LCUs by analyzing to what extent their use can promote adverse events on materials and patients’ oral condition when compared to low-power LED-LCUs, on daily dental practice. PubMed and B-on database search focused on high-power (≥2000 mW/cm^2^) LED-LCUs outputs. Studies assessing performance of high-power LED-LCUs for light-curing dental-resin-based-materials were included. From 1822 screened articles, 21 fulfilled the inclusion criteria. Thirty-two marketed units with high levels of radiant emittance (≥2000 mW/cm^2^ up to 6000 mW/cm^2^) were identified. Most output values vary on 2000–3000 mW/cm^2^. The highest output found was 6000 mW/cm^2^, in FlashMax^™^P3. Reports suggest that light-curing protocols with lower emittance irradiance and longer exposure outperforms all other combination, however in some clinical procedures high-power LED-LCUs are advocated when compared to low-power LED-LCUs. Moreover, long time exposures and over-curing can be dangerous to the biological vital pulp, and other oral tissues. Evidence showing that high-power LCUs are the best clinical option is still very scarce.

## 1. Introduction

Light-curing units (LCU) and an efficient irradiation procedure is indispensable for the clinical success of resin-based dental materials; thus, being aware of this simple, perhaps important task is crucial. Clinicians have at their disposal an unprecedented variety of LCUs, so, in order to make informed decisions, dentists need to consider if the device they are operating is emitting optimal light parameters.

The quantity and quality of light generated by a LCU is highly dependent on the radiant emittance, exposure time, and spectral emission, respectively, but also on the interactions between these variables and their compatibility with the monomeric properties of the restorative material [1].

Radiant emittance (expressed in units of mW/cm^2^) describes the output from a curing light device and therefore is the power emitted by the light source divided by the area of the light tip. While irradiance—also referred to as intensity or power density—is the power incident on a surface and describes the quantity of light-energy that a resin-based material receives (also expressed in units of mW/cm^2^) [2]. The International Organization for Standardization (ISO) 4049:2000 considered an irradiance level of 300 mW/cm^2^ as being enough to induce adequate polymerization of light-cured resin-based materials [1].

Spectral emission is designated by the wavelength range within the electromagnetic spectrum emitted. Most dental resin-based materials contain two-components: the photo-initiator, generally a camphorquinone (CQ) or other photo-initiator systems as diphenyl phosphine oxide (TPO) and as Ivocerin [3] which can absorb light directly, and a co-initiator, typically an amine, that does not absorb light but interacts with the activated photo-initiator to generate a reactive free radical and initiates resin-monomers polymerization. Efficient monomer polymerization occurs when corresponding wavelengths match the maximum absorption of the material’s photoinitiator system [4,5].

Nonetheless, the total delivered optical energy depends on the exposure time. The emission of light to a given surface over time is called radiant exposure (expressed in units of J/cm^2^) [6]. Current light-emitting diode (LED)-LCUs may have multiple light modes with variation on light output emission and time protocol, allowing different radiant exposures.

A critical feature of the light-cured composites and every resin-based dental material is their adequate polymerization. The degree of monomer polymerization is determined by the proportion of the remaining concentration of the double carbon bonds in a polymerized sample relative to the total number of double carbon bonds in the uncured material. The degree of conversion directly affects the chemical, physical, and mechanical properties of resin-based composites (RBC) and a higher degree of conversion indicates a greater amount of dental material monomer polymerization [7].

Since LED-LCUs were marketed and introduced, their use became increasingly popular for curing dental materials.

Nevertheless, it was not before the 1990 s, when the first commercial LED-LCU became available, that LEDs were seriously considered by scientists or manufacturers of commercial LCUs as light sources to photopolymerize dental composites and other dental materials [8].

First generation LED’s radiant emittance ranged from 160 to 400 mW/cm^2^, implying less curing potential than conventional competitive types of LCUs.

Later, in 2002, a second generation with a higher radiant emittance range of 500–1400 mW/cm^2^ was introduced, being able to reach values up to 1500 mW/cm^2^ [9,10]. Those versions emitted a similar narrow spectrum designed to match the absorption spectrum of CQ, the most common photoinitiator of resin-based dental materials composition [8].

Around 2004, the advances in LED technology enabled the development of higher power LED-based systems: the third generation of LED curing units [8]. The radiant emittance levels were increased, and those medical devices are now capable of delivering radiant emittance values up to 5000 mW/cm^2^ [11]. Additionally, in order to address the mismatch between the emission spectrum and photoinitiators besides CQ, an additional emission peak was introduced in LED-LCUs, able to activate a wider range of photoinitiators. Those are equipped with multiple diodes, able to radiate both violet and blue light having an optimal spectral wavelength range within 400–500 nm (nanometers) [8,12].

Newer LED-LCUs are currently being introduced on the market, advocating a fourth-generation technology. Among several enhancements, a wavelength scanning technology is the most significant improvement, allowing the clinician to select the appropriate output mode according to the material and the clinical condition [13].

Light-curing technology continues to evolve at a rapid pace. From the first generation of LED-LCUs that produced poor output, to the current third generation that became capable of delivering higher radiant emittance in short exposure time, these enhanced devices also became polywave, emitting multiple wavelengths bands [8,12].

High-powered LCUs have increased radiant emittance and claimed it could be used for shorter exposure time, thus reducing the curing time needed for dental resin-based materials. However, there is still insufficient research regarding the resin-based materials clinical survival rates and polymerization quality by the newly developed high-power LED-LCUs. Low rate of monomers-to-polymer degree of conversion is undesirable, and promotes low chemical, physical, and mechanical properties of resin-based materials in short- and mean-term clinical environments. Material deterioration and subsequent increasing water absorption, marginal wear, microleakage, discoloration, staining and [14,15,16,17,18,19] also monomer elution and leaching out of components to the pulp or gingival tissues and decrease in material biocompatibility may occur [15]. Also, data regarding clinical adverse events those LCUs may promote in dentin-pulp complex/biological tissues is another clinical condition to be addressed. The consecutive or prolonged light-curing mode emissions induce significant and cumulative temperature rise, leading to a potential damage of the dentin-pulp complex [20,21,22]. Symptoms such as hyperalgesia, hypersensitivity, and spontaneous pain, typical of acute pulpitis, suggest this damage [21]. As claimed by Rueggeberg et al. and Alasiri et al. other adverse events have been reported, such as burning sensation on the surrounding soft tissues and ocular hazard, when high levels of blue light are emitted [23,24].

Therefore, the aim of this review is to describe the current high-power LED-LCUs, including a bibliometric systematization of in vitro and in vivo studies of LED-LCU parameters and possible adverse events that can occur, both on resin-based dental materials and patients’ oral condition, while applying it to daily dental practice.

## 2. Materials and Methods

This review was conducted in accordance with the guidelines of an integrative review and the research question, based on the PICO model, aimed to assess the current knowledge on dentistry-based high-power LED-LCU currently available (Problem) by analyzing to what extent their use (Intervention) can promote adverse events on materials and patients’ oral condition (Outcome), in in vitro and in vivo assays, when compared to low-power LED-LCU (Control), used in daily dental practice.

### 2.1. Inclusion and Exclusion Criteria

Only studies that evaluated irradiation protocols with high-power output devices were included. In Vitro and in vivo studies assessing the performance parameters of high-power LED-LCU currently available and assessing any adverse events on materials and patients’ oral condition were included. Performance parameters of high-power LED-LCU were established to devices with radiant emittance values ≥2000 mW/cm^2^. Only articles written in the English language and published within the years 2010 to 2021 were contemplated for this review. Articles reporting exclusively halogen, plasma-arc, or argon-ion laser curing lights, light-cured materials polymerized with radiant emittance below 2000 mW/cm^2^ or whose radiant emittance was not mentioned were excluded.

The inclusion and exclusion criteria were established by a consensus reached from two examiners (RA and PMM) after discussion, considering the research question and the objectives of the study while aiming for an ample range of results to be provided from the search.

### 2.2. Search Strategy

#### 2.2.1. Sources of Information

An electronic search was made in PubMed and B-on electronic databases. The structured search strategy of the articles and data extraction were conducted by two calibrated examiners (RA and PMM) in order to identify all in vivo and in vitro studies on high-power LED light-curing units.

To collect data of the commercially available LCUs used in dental offices an extensive on-line hand-search was conducted. The last search was performed in April 2020. Manufacturers were identified through the 2019 exhibitors’ manual of an international dental congress (Lisbon, Portugal). All manufacturers were assessed (online site) in order to identify all commercially available LED-LCUs with a claimed output ≥2000 mW/cm^2^. Additionally, two dental material manufacturer’s devices mentioned in the reviewed studies, that met the inclusion criteria, were retained for quality assessment.

If any information was missing, the manufacturers were contacted via e-mail to supply the missing data. When manufacturers failed to provide an answer, the respective information was mentioned as “Not Found”.

#### 2.2.2. Search Terms

The search strategy included 5 Mesh (Medical Subjects Headings) terms: “Dental Curing Units”, “LED Dental Curing-Lights”, “Composite Resins”, “adverse events” “dental materials” and 11 uncontrolled descriptors: “LED dental curing-lights”, “dental curing lights“, “curing lights dental”, “high-power LED”, “high intensity LED”, “resin-based composite”, “dental resins”, “dental materials”.

Boolean operators (“OR” and “AND”) were used to join search terms relevant to the search question (Table 1). The last search was performed in March 2021.

#### 2.2.3. Study Selection

Articles identified using the search terms were exported to Mendeley desktop 1.19.4 software to check for duplicates. A first screening of record titles and abstracts was carried out by two independent examiners according to the inclusion and exclusion criteria. The remaining studies were assessed for eligibility and qualitative synthesis by full-text screening.

#### 2.2.4. Study Data

Bibliometric analysis was performed recording the following variables: authors and year of publication. The methodology of analysis included the summary of aims, materials and methods and outputs of the included studies, by transcription of the following variables: study type, clinical procedure performed, high-power LED-LCU used, radiant emittance (by manufacturer and used in the study), exposure time, control LCU used and respective radiant emittance and exposure time, and lastly the irradiation technique employed.

For the synthesis of outcomes, studies were categorized in terms of significant results found regarding the LCU used and/or the adverse events that occurred on resin-based dental materials and patients’ oral condition, by their use.

Additionally, for the synthesis of identified high-power LED-LCUs currently and commercially available, manufacturer’s LCU Directions for Use (DFU) were assessed, and the following variables were transcribed: manufacturer, LED-LCU, high-power curing modes’ radiant emittance and exposure time, estimated wavelength range, incorporated (yes/no) light meter and link to DFU.

### 2.3. Quality Assessment

The quality of the studies was assessed using the modified CONSORT checklist of items for reporting pre-clinical studies on dental materials/devices [25] and the CONSORT 2010 guidelines for reporting randomized clinical trials [26].

## 3. Results

### 3.1. Study Selection and Flow Diagram

**Figure 1:**
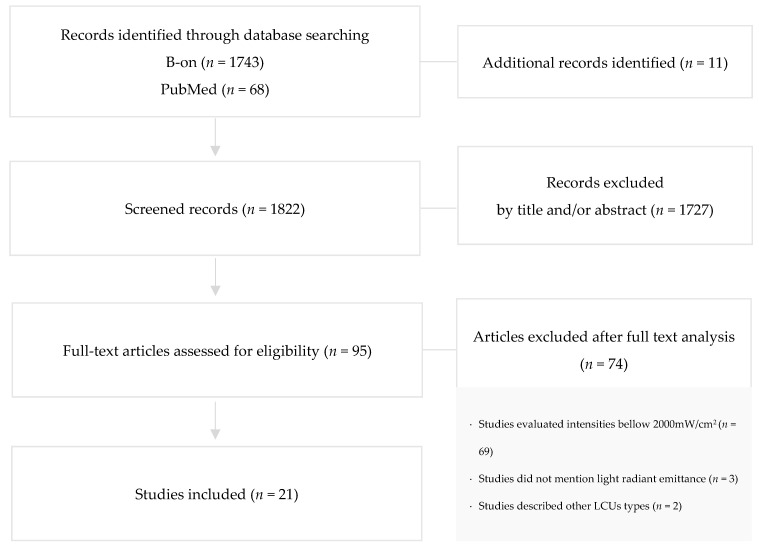
Search flowchart according to PRISMA illustrating study inclusion.

A total of 1822 preliminary references related to dental articles were assessed (Figure 1).

After excluding duplicates, the remaining articles were screened and 1727 were excluded by reading the title and/or abstract. The resulting 95 articles were examined at full-text level, and out of these, 74 articles were excluded due to evaluation of intensities bellow 2000 mW/cm^2^ (*n* = 69), unmentioned light radiant emittance (*n* = 3), and description of other LCUs type (*n* = 2).

### 3.2. Study Characteristics

#### 3.2.1. Study Design

To evaluate the performance of LED-LCUs with radiant emittance ≥2000 mW/cm^2^ a total of 21 articles were considered: 17 in vitro and 3 in vivo studies. Additionally, 1 article presented both an in vitro and an in vivo setting. All reviewed studies are summarized in Table 2.

#### 3.2.2. Clinical Procedure

High-power LED-LCUs studied were assessed in several daily clinical procedures. They were analyzed to determine what extent their use can promote adverse events on teeth submitted to irradiation protocols and on several dental resin-based materials such as resin cements, sealants, resin-based composites, and adhesive systems.

#### 3.2.3. High-Power LED-LCUs

High-power LED-LCUs evaluated ranged from commercially available to experimental devices. Radiant emittance of high-power curing modes according to the manufacturer and mean values measured in the studies were presented. Exposure time applied was also included.

#### 3.2.4. Control LCUs

Quartz tungsten halogen LCUs, standard LED-LCUs or high-power LED-LCUs in standard mode were used to compare the results. Radiant emittance according to the manufacturer and exposure time applied were considered.

#### 3.2.5. Irradiation Technique

Only 14 studies mentioned the irradiation protocol used. One study stated it was followed the LCU manufacturer’s protocol without specifying the procedure [27]. Thirteen studies reported in detail the irradiation technique applied [10,11,14,16,17,21,22,28,29,30,31,32,33].

**Table 2:** Summary of the reviewed studies.

Author, Year	Study Design	Clinical/ Technical Procedure	High-Power LED-LCU (Manufacturer)	Radiant Emittance ^1^ (mW/cm^2^)	Exposure Time ^1^ (s)	Radiant Emittance Mean Value ^2^ (sd) (mW/cm^2^)	Control LCU (Manufacturer)	Radiant Emittance (mW/cm^2^)	Exposure Time (s)	Irradiation Technique
Park, Roulet and Heintze, 2010 [22]	In Vitro	Maxillary premolar exposed to light curing units	**LED_exp2000_ Prototype** (Ivoclar Vivadent, Liechtenstein) **LED_exp3000_ Prototype** (Ivoclar Vivadent, Liechtenstein)	2000 3000	60 60	Not reported	Astralis^®^ 10 (Ivoclar Vivadent, Liechtenstein) Bluephase^®^ 16i (Ivoclar Vivadent, Liechtenstein)	1200 1600	30 60	The unprepared tooth was light cured from the buccal side 1 mm from the buccal surface.
Flury et al., 2013 [10]	In Vitro	Light curing resin cements through glass ceramics	**VALO^®^** (Ultradent, Utah, USA)	3200	18	3505	Elipar^™^ Freelight 2 (3M ESPE, Seefeld, Germany) VALO^®^ (Ultradent, Utah, USA)	1200 1400	40 32	Light curing was performed either directly or through ceramics discs of 1.5 or 3 mm.
Branchal et al., 2015 [31]	In Vitro	Light curing sealants	**VALO^®^** (Ultradent, Utah, USA) **Fusion^®^** (DentLight Inc., Texas, USA) **SmartLite^®^ Max** (Dentsply International, York, PA, USA)	3200 2700 2805	3, 6, 9 5, 10 10	3539 2773 2012	3M^™^ XL 3000 (3M ESPE, Minn, USA)	450	40	The light tip was placed directly in contact with the surface.
Gonulol, Ozer and Tunc, 2015 [16]	In Vitro	Polymerization of resin-based tooth-colored restorative materials	**VALO^®^** (Ultradent, MO, USA)	3200	6	Not reported	Elipar^™^ S10 (3M ESPE, MN, USA) VALO^®^ (Ultradent, MO, USA)	1200 1000 or 1400	10 20 or 12	The light guide was in contact with the top surface.
Haenel et al., 2015 [17]	In Vitro	Light curing dental resins	**Bluephase^®^ 20i** (Ivoclar Vivadent, Schaan, Liechtenstein)	2200	5, 20, 80	2222	Celalux^®^ 2 (VOCO, Cuxhaven, Germany) Bluephase^®^ 20i (Ivoclar Vivadent, Liechtenstein)	1000–1500 ^3^ 650	5, 20, 80 5, 20, 80	The light guide tips were placed directly over the center of the sample.
Runnacles et al., 2015 [32]	In Vivo	Human premolars exposed to a light curing unit	**Bluephase^®^ 20i** (Ivoclar Vivadent, Schaan, Liechtenstein)	2000	5	2204 (35)	Bluephase^®^ 20i (Ivoclar Vivadent, Liechtenstein)	650 1200	10 10, 60	The LCU tip was placed against the buccal tooth surface with the lower edge of the light guide sheath just above the facial free gingiva.
Ward et al., 2015 [34]	In Vivo	Brackets cured with a high-intensity LED	**VALO^®^ Ortho** (Ultradent, UT, USA)	3200	6	Not reported	VALO^®^ Ortho (Ultradent, UT, USA)	1200	20	Not reported
Watanabe et al., 2015 [33]	In Vitro	Polymerization of dual-cured resin cement	**PenCure 2000** (Morita, Tokyo, Japan) **VALO^®^** (Ultradent, Utah, USA)	2000 3200	5, 10, 15, 20 5, 10, 15, 20	Not reported	Jetlite 3000 (Morita, Tokyo, Japan) Demi^™^ (Kerr, CA, USA)	400 1100	20, 40, 60, 80 20, 40, 60, 80	The tip of the curing unit was placed on the top of the ceramic plate.
Armellin et al., 2016 [20]	In Vitro	Composite restorations in first molars	**VALO^®^** (Ultradent, Utah, USA)	3200	3	1600	VALO^®^ (Ultradent, Utah, USA) Starlight PRO (Mectron, Carasco, Italy)	1000 1000	20 20	Not reported
Oz, Oz and Arici, 2016 [35]	In Vivo/ In Vitro	Metal brackets polymerized intraorally and, to extracted tooth	**VALO^®^ Ortho** (Ultradent, Utah, USA)	3200	3	Not reported	Elipar^™^ S10 (3M Unitek, Monrovia, Calif)	1600	10	Not reported
Peutzfeldt, Lussi and Flury, 2016 [28]	In Vitro	Light curing resin cements	**VALO^®^** (Ultradent, UT, USA)	3200	3 6 9	3162 (88.2) 3213 (110.9) 3299 (89.0)	VALO^®^ (Ultradent, UT, USA)	1000 1400	10, 20 8, 16	The tip end of the LCUwas placed at a distance of 0 mm.
Bilgic et al., 2017 [27]	In Vitro	Curing of orthodontic adhesives	**VALO^®^ Ortho** (Ultradent, Utah, USA)	3200	3	Not reported	VALO^®^ (Ultradent, Utah, USA)	1400	3	According to the manufacturer’s instructions.
Shimokawa et al., 2017 [15]	In Vitro	Polymerization of resin-based composites	**Single-peak high Prototype** (Ultradent, UT, USA) **Broad-spectrum high Prototype** (Ultradent, UT, USA)	3600 3600	5 5	3607 (16.6) 3612 (26.0)	Single-peak standard Prototype (Ultradent, UT, USA) Broad-spectrum standard Prototype (Ultradent, UT, USA)	1200 1200	15 15	Not reported
Udomthanaporn, Nisalak and Sawaengkit, 2017 [11]	In Vitro	Orthodontic brackets bonded to human premolars	VALO^®^ (Ultradent, UT, USA) **FlashMax^™^ P3** (CMS Dental, Copenhagen, Denmark)	3200 4000–6000	6 3	Not reported	Bluephase^®^ (Ivoclar Vivadent Inc., Amherst, NY, USA)	1200	20	The tip of each curing unit was held about 1 mm away from the bracket-tooth interface.
Almeida, Martins and Martins, 2018 [18]	In Vitro	Bracket bonding to human premolars	**VALO^®^ Cordless** (Ultradent, UT, USA)	3200	3	2246	VALO^®^ Cordless (Ultradent, UT, USA)	3200	6	Not reported
Daugherty et al., 2018 [14]	In Vitro	Polymerization of bulk-fill composites	**FlashMax^™^ P3** (CMS Dental, Copenhagen, Denmark) **S.P.E.C.^®^ 3** (Coltene, OH, USA)	5000–6000 3000–3500	3, 9 3	2378 (22) 3001 (8)	Paradigm^™^ (3M ESPE, MN, USA) S.P.E.C.^®^ 3 (Coltene, OH, USA)	1200 1600	10, 20 20	The LCU light tip was positioned concentrically to the mold opening and directly against the surface.
Nurlatifah, Eriwati and Indrani, 2018 [19]	In Vitro	Curing of packable composite resin	**FlashMax^™^ P3** (Hexagon, Denmark)	4000	1, 3	1200	Ledmax^™^ 450 (Hilux, Benlioglu Dental Inc., Ankara, Turkey)	450	20	Not reported
Vinagre et al., 2019 [21]	In Vitro	Immediately extracted premolars submitted to light curing procedures	**Bluephase^®^ 20i** (Ivoclar Vivadent, Schaan, Liechtenstein) **S.P.E.C.^®^** **3** (Coltene, OH, USA)VALO^®^ (Ultradent, UT, USA)	2000 3000 3200	5 3 3	1790 2420 2710	Bluephase^®^ 20i (Ivoclar Vivadent, Liechtenstein) S.P.E.C.^®^ 3 (Coltene, OH, USA) VALO^®^ (Ultradent, UT, USA) Demi Ultra^™^ (Kerr, Orange, CA, USA)	1200 1600 1000 or 1400 ^4^ 1215 or 1100–1330 ^4^	20 15 20 or 4 ^4^ 20 or 20 ^4^	The LCUs were placed in a support with the light guide touching the buccal surface of the teeth and four light emissions were made with 30 s intervals.
Gross et al., 2020 [29]	In Vivo	First premolars requiring extraction exposed to a Polywave LED LCU	**Experimental LCU** (Ivoclar Vivadent, Schaan, Liechtenstein)	10,000	1,2	10,000	Bluephase^®^ 20i (Ivoclar Vivadent, Liechtenstein)	1200	10, 20, 60	LCU tip was placed against the buccal tooth surface, directly centered over the Class V preparation.
Sadeghyar, Watts and Schedle, 2020 [30]	In Vitro	Ultra-fast polimerization of bulk-fill resin-based composite RAFT-modified	**Bluephase^®^ Power Cure** (Ivoclar Vivadent, Schaan, Liechtenstein)	3000	3	3770.3 (±35.30)	Bluephase^®^ Power Cure (Ivoclar Vivadent, Schaan, Liechtenstein)	1200	10	The LCU was placed perpendicular, directly on the top surface of the cylinder.
Rocha et al., 2021 [36]	In Vitro	Light-curing of a bulk fill composite	**VALO^®^** (Ultradent, UT, USA)	3200	9	2244	VALO^®^ (Ultradent, UT, USA)	1000	21	Not reported

^1^ According to the manufacturer. ^2^ Measured in the study—mean value (standard deviation). ^3^ Depending on the light tip. ^4^ Depending on the mode.

### 3.3. Quality Assessment

All in vitro studies analyzed with the modified CONSORT checklist (Table 3) presented a structured abstract (item 1) and an introduction which provided scientific background about light-curing in dentistry (item 2a) and clear objectives and hypotheses (item 2b). Description of methodology as well as of the variables studied was sufficiently clear to allow for replication in all studies (items 3 and 4), but the majority of them did not present a detailed report of the calculation of sample size or random allocation sequence (items 5–9). All studies indicated the statistical method used (item 10), but presented significance level as p values, and not confidence intervals (item 11). Discussions included a brief synopsis of the key findings, comparisons with relevant findings from other published studies and limitations of the studies (item 12). Sources of funding (if any) were indicated in the majority of studies (item 13), and indications for access to full trial protocols were obviated in all studies (item 14).

The four in vivo studies analyzed using the CONSORT 2010 guidelines (Table 4) were not identified as a randomized trial in the title (item 1a) but all provided a clear and detailed abstract (item 1b). In the introduction all the authors presented specific scientific background of the high-power LED-LCUs studied (item 2a) and established the purposes of the trial (item 2b).

Details about the trial design and conceptual framework were not clearly described in all studies (item 3a) but information about the eligibility criteria (item 4a), settings and locations where the data were collected (item 4b) and intervention for each group (item 5) were included in all studies. No deviations from the protocol were made and reported during the course of the trials (item 3b an item 6b). Completely defined pre-specified primary and secondary outcome measures, including how and when they were assessed, were presented (item 6a). No interim analysis was performed (item 7b). One study failed to mention how sample size was determined but the remaining authors stated the ideal sample size for detecting clinical statistically significant results (item 7a), nevertheless, none of the studies presented a detailed report of the random allocation sequence and implementation (items 8a–11b). Both statistical methods and additional analyses (item 12a and item 12b) were shown in all studies. Although all studies reported for each group the number of participants who were randomly assigned, received intended treatment, and were analyzed for the primary outcome (item 16), no participant flow diagram was included for analysis (item 13a and 13b); none had a table presenting baseline demographic and clinical characteristics for each group (item 15). All of the studies included the periods of follow-up but two failed to report the dates of recruitment (item 14a). No decision to early stop the trial was reported in any study (item 14b). The results for each study group were reported in all studies but were carried out with *p* values as a measure of precision and not confidence intervals (item 17a and 17b). Moreover, no additional analyses of the same data were undertaken (item 18). The studies did not mention any unintended adverse effects each group may have suffered (item 19). Items referring to the discussion were fulfilled by all studies (items 20–22). Lastly, information as registration number and where the full trial protocol can be accessed were obviated in all cases (item 23 and 24) and sources of funding (if any) were described (item 25).

### 3.4. Study Results

A list of the main adverse events described in the included studies are presented in Table 5. Sixteen studies investigated the effects on dental resin-based materials’ properties cured with high-power LED-LCUs: five directly measured the degree of conversion, two assessed the depth of cure, and eight analyzed microhardness values. Other properties were evaluated, such as diametral tensile strength, elastic modulus, and bond strength. Five studies aimed to evaluate consequences to the patient, particularly temperature changes in the pulp exposed to high-power LED-LCUs.

The analysis of the tests carried out and outcomes of the main adverse events described in the studies are summarized in Table 6.

#### 3.4.1. Degree of Conversion (DC) Analysis

This property can be assessed by several methods. Five studies directly examined the degree of conversion (DC) of resin-based dental materials through Fourier transform infrared spectrometry (FT-IR). A study by Shimokawa et al. used a custom-designed LCUs—a Single-peak high Prototype and a Broad-spectrum high Prototype both emitting 3600 mW/cm^2^ for 5 s and a Single-peak standard Prototype and a Broad-spectrum standard Prototype (Ultradent, UT, USA) equally emitting 1200 mW/cm^2^ for 15 s—with the same light tip and construction allowing better standardization of the light conditions. The four emission conditions delivered similar radiant exposures. The authors reported that compared to 3600 mW/cm^2^, equivalent or higher DC values were achieved using 1200 mW/cm^2^ radiant emittance [15]. In the Flury et al. study, VALO^®^ and Elipar^™^ Freelight^2^ LCUs were used, selecting light-curing times longer than the recommended by the manufacturers to achieve identical radiant exposures when polymerizing resin cements. Outputs of this study showed that a higher irradiance generally did not result in higher DC within a given resin cement. Similar DC were achieved, though at shorter curing times [10]. Haenel et al. studied Bluephase^®^20i and Celalux^®^2 LCUs, delivering an irradiance of 666 mW/cm^2^, 2222 mW/cm^2^, and 1264 mW/cm^2^ for 5, 20, and 80 s respectively, to dental resins. The final DC after 3 min of measurement exhibited higher DC values for the *Turbo* mode (2222 mW/cm^2^) than for the *Low* mode (666 mW/cm^2^) specimens. For example, in the *Turbo* mode an exposure time of 5 s lead to a DC (3 min) of 62.5% while after 80 s a DC of 67.3% was reach. In the *Low* mode the corresponding DC (3 min) values were 54.2% and 65.2%, respectively [17]. Daugherty et al. compared different light-curing combinations on bulk-fill composites. S.P.E.C.^®^3 in *3* s *mode* and FlashMax^™^ P3 were categorized as the high irradiance group (≥2000 mW/cm^2^) and S.P.E.C.^™^3 in *main mode* and Paradigm^™^ as the conventional irradiance group (≤2000 mW/cm^2^). It also categorized *Standard*, a *Short,* and *Ultra-short* exposure times to be of 20 s, 9–10 s, and 3 s, respectively. The authors concluded that radiant exposure—the product of irradiance and exposure time—is more correlative to DC than the irradiance parameter itself, and that the polymerization protocol of bulk-fill composites with *standard-irradiance & long-exposure* time outperforms all other combinations [14].

Assessment of depth of cure and microhardness testing were also used to appraise the effect of different irradiance emittances. Depth of cure was evaluated by Daugherty et al. and results reveled that the curing combination of *standard irradiance & exposure* was not significantly different than the *high irradiance & short exposure* combination. However, the curing combination of *standard irradiance & exposure* significantly outperformed *standard irradiation & short exposure* and *high irradiance & ultra-short exposure* combinations [14]. The hardness (KHN value) parameter was examined in seven studies [15,16,17,27,28,31,33]. In accordance with DC values, equivalent or higher degree of microhardness values of resin composites were achieved when 1200 mW/cm^2^ was used compared to 3600 mW/cm^2^ [15]. Gonulol et al. light-cured a microhybrid composite resin, a giomer-based composite resin, a compomer and a resin-modified glass ionomer cement with different irradiance emittances (*Standard*—1200 mW/cm^2^; *High-power*—1400 mW/cm^2^; *Extra-power*—3200 mW/cm^2^) of VALO^®^ and used Elipar^™^ S10 LCU as control. VALO^®^ used in *Extra-power* mode for 6 s did not achieve sufficient polymerization of the restorative material, however when applied for 12 s the *High-power* mode achieved microhardness values similar to those obtained with the VALO^®^ in *Standard* mode and Elipar^™^S10. Authors concluded that *High-power* mode of the VALO^®^ can be recommended for clinical applications as it can shorten the time required to properly polymerize resin-based restorative materials [16]. In the study by Haenel et al., when using the Bluephase^®^20i and Celalux^®^2 for 5 s the mean hardness value of the surface of dental resins increased with exposure time [17]. Branchal et al. light-cured three sealants (opaque-unfilled, opaque-filled, and clear-filled). The shortest exposure time recommended by the manufacturers (VALO^®^ for 3 s; Fusion^®^ for 5 s; SmartLite^®^ Max for 10 s) was doubled or tripled, without exceeding the manufacturer’s longest exposure limit. A halogen LCU was used for 40 s, as control. The authors reported that opaque-filled and clear-filled sealants hardness values were statistically equivalent or better when light-cured with VALO^®^ for 6 or 9 s than the control LCU, at a depth of 1.5 mm. Fusion^®^ LCU for 10 s did not adequately cure the three sealants beyond 1 mm. SmartLite^®^ LCU for 15 s did not adequately cure the sealants beyond 0.5 mm [31]. Bilgic et al. evaluated the effects on orthodontic adhesives when applying 1400 mW/cm^2^ or 3200 mW/cm^2^ with VALO^®^. Higher hardness values were achieved in the adhesives cured with 3200 mW/cm^2^ for 3 s. KHN increased as the irradiation time was extended in the Watanabe et al. study. The authors concluded that when polymerizing dual-cured resin cements, through ceramic material, high-intensity LED units required a shorter irradiation period than halogen and standard LED-LCU to obtain KHN similar to those observed during direct irradiation [33]. Peutzfeld et al. also studied the impact on KHN of resin cements. Three dual-curing resin cements and a flowable resin composite were light-cured with VALO^®^ in *Standard* mode (1000 mW/cm^2^), *High power* mode (1400 mW/cm^2^), or *Xtra power* mode (3200 mW/cm^2^). Distinct exposure times were set to obtain two or three levels of radiant exposure, in each light-curing mode. Authors concluded that high irradiance light-curing modes do not impact polymerization of resin-based materials [28].

#### 3.4.2. Diametral Tensile Strength (DTS) Analysis

Only one study focused on this subject [19]. Specimens of packable composite resin were cured with FlashMax^™^ P3 and Ledmax^™^450 LCUs. The group of specimens that received a high amount of total light energy had high DTS, while the group receiving low total light energy showed lower DTS. The group cured with Ledmax^™^450 for 20 s had the highest DTS compared to the two other groups cured with FlashMax^™^P3 for 1 and for 3 s. The authors concluded that the curing modes influence the DTS of packable composite resin [19].

#### 3.4.3. Elastic Modulus (EM) Analysis

Bilgic et al. studied the elastic modulus of adhesives in orthodontics and reported higher elastic modulus values when VALO^®^ was applied at 3200 mW/cm^2^ mode for 3 s than when applied at 1400 mW/cm^2^ for 3 s [27].

#### 3.4.4. Bond Strength (BS) Analysis

In order to determine BS, clinical bond failure rates were examined in two in vivo studies [34,35]. Ward et al. selected 34 patients and a total of 680 brackets were bonded using a randomized split-mouth design. In this study two different settings of VALO Ortho^®^ were used. In 17 participants the maxillary right and mandibular left quadrants were cured with 3200 mW/cm^2^ setting for 6 s per tooth while the maxillary left and mandibular right quadrants were cured for 20 s with 1200 mW/cm^2^. On the other 17 patients the quadrants were inverted. All participants were observed for a minimum period of 6 months. The brackets bond failure rate was 1.18% for both curing methods. The authors concluded that 6 s curing time per tooth with a high-power curing light is sufficient to produce clinically adequate bond failure rates, that are comparable to brackets cured with a standard LED-LCU for 20 s [34]. In Oz et al. clinical trial, 40 participants were included, and a split-mouth design was applied. In group 1, the adhesive was cured for 10 s (1600 mW/cm^2^) with Elipar S10^™^ and in group 2 for 3 s (3200 mW/cm^2^) with VALO Ortho^®^. Bond failure rate at 12^th^ month were 2.90% and 3.16%, respectively, allowing to conclude that bracket bonding can be safely accomplished with the two LED-LCUs [35].

Additionally, in vitro bond strength was determined evaluating shear bond strength (SBS) values [11,18,35] and the adhesive remnant index (ARI) [11]. Oz et al. compared the performance of Elipar S10^™^ and VALO Ortho^®^ LCUs by bonding brackets to extracted premolars, using the same curing times. No significant difference on bond strengths (9.8 ± 4.27 MPa and 11.43 ± 3.56 MPa) and ARI score were found between groups [35]. When comparing two exposure times while applying the same LED-LCU and the same radiant emittance, Almeida et al. achieved a significantly higher SBS using a 6 s interval instead of 3 s interval, concluding that time significantly affected SBS values. The 3 s interval produced an average SBS of 15.79 MPa while the 6 s interval produced an average of 21.57 MPa. The use of high-power LED reducing the curing time from 6 to 3 s decreased the bond strength. [18]. Udomthanaporn et al. light-cured adhesives with Bluephase^®^ (1200 mW/cm^2^ for 20 s), VALO^®^ (3200 mW/cm^2^ for 6 s) and FlashMax^™^P3 (4000–6000 mW/cm^2^ for 3 s). Similar mean SBS was found for Bluephase^®^ (21.80 ± 2.85 MPa) and VALO^®^ (21.04 ± 2.87 MPa); significant lowest mean SBS (4.75 ± 2.82 MPa) was found for FlashMax^™^P3, despite the highest radiant emittance [11].

#### 3.4.5. Temperature Changes in Pulp Tissue Analysis

The increase of temperature in pulp tissue during the light-curing process was assessed in five studies, by Optical Fibber Bragg grating sensors [21] or different types of thermocouples [20,22,29] inserted into the pulp chamber of extracted teeth, or intraorally but requiring tooth extraction after.

Runnacles et al. performed an in vivo study in eight volunteers with well-controlled health conditions requiring extraction of healthy, intact, non-carious, non-restored, fully erupted, upper right and left premolars that were sequentially exposed for 10 s in *low intensity* (656 mW/cm^2^), 10 s in *high intensity* (1244 mW/cm^2^), 60 s in *high intensity* (1244 mW/cm^2^) and 5 s in *turbo intensity* (2204 mW/cm^2^), with Bluephase 20i^®^. The lowest variation in pulp temperature was reveled for 10 s in *low intensity* (656 mW/cm^2^) mode; on the other hand, 60 s in *high intensity* (1244 mW/cm^2^) mode resulted in the highest peak and variation in temperature, with some pulp chambers exhibiting a temperature rise above 5.5 °C. No significant difference for the peak pulp temperature was observed for *5* s/*turbo intensity* (2204 mW/cm^2^) and 10 *s/high intensity* (1244 mW/cm^2^) modes [32]. Armellin et al. showed that longer exposure times (20 s in 1000 mW/cm^2^) resulted in higher increase of pulp tissue temperature, when comparing to 3 s in 3200 mW/cm^2^, although radiant emittance was lower [20]. Park et al. registered a temperature increase from 41.0 °C to 53.7 °C depending on the LCU used. Considering 34 °C as the temperature of pulp tissue, an increase of 17 °C and of 19 °C, occurred after 60 s of exposure to the LED_exp2000_ Prototype and the LED_exp3000_ Prototype LCU, respectively. Intra-pulpal temperature increased more than 5 °C when the exposure time was longer for more than 10 s with the LED_exp2000_ Prototype and the LED_exp3000_ Prototype [22]. Vinagre et al. concluded that high-energy-level curing modes produced pulp temperature variations around or above 5.5 °C [21]. Gross et al. compared specific scenarios that represented near similar radiant exposures (10 s with 1231 mW/cm^2^ by Bluephase 20i^®^ and 1 s with 10,000 mW/cm^2^ by the experimental LED-LCU—12.3 and 10.0 J/cm^2^, respectively—and 20 s with 1231 mW/cm^2^ by Bluephase 20i^®^ and 2 s with 10,000 mW/cm^2^ by the experimental LED-LCU—24.6 and 20.0 J/cm^2^, respectively). The authors concluded that there is no significant difference in pulp temperature changes between 20s/20i and 2s/EXP groups which in turn exhibited significantly higher temperature changes values than 1s/EXP and 10s/EXP [29].

Temperature pulp changes after LED-LCU emission were also observed. Runnacles et al. study revealed that the pulp temperature still remains increased for a few seconds, and then slowly decreases to the pre-exposure baseline value, taking approximately 4 to 5 min to this condition [32]. According to Park et al., the subsequent decrease in pulp temperature was 0.16 °C/seconds and 0.24 °C/seconds, after turning off the 30 s exposure time with LED_exp2000_ Prototype and LED_exp3000_ Prototype, respectively [22]. Vinagre et al. evaluated the pulp temperature changes after four consecutive exposure modes. During the 30 s rest periods between light emissions, pulp temperature did not recover to baseline levels. Instead, pulp temperature continued to increase until a plateau was reached about halfway through each rest period. Afterwards, pulp temperature decreased approximately 1 °C until a new light emission started [21]. Similar behavior was noticed in the study by Gross et al. where the pulp temperature kept increasing after the curing light shut off, followed by a rapid drop and slower decrease until the baseline pulp temperature was reestablished.

#### 3.4.6. High-Power LED-LCUs Commercially Available

Thirty-two marketed LED-LCU with high power radiant emittance were identified and their technical details described in Table 7.

The manufacturers BA International (Northampton, UK), Clarben (Madrid, Spain), Coltene Iberia S.L. (Madrid, Spain), DiaDent Europe (Almere, The Netherlands), Morita Europe (Dietzenbach, Germany) and MyRay (Bologna, Italy) commercialize each, only one high-power LED-LCU.

On the other hand, Bader (Nigrán, Spain) developed distinct high-power units.

Aiming to incorporate particular features (i.e., broader emission spectrum or heads with different diameters) Acteon (Merignac, France), CMS Dental (Copenhagen, Denmark), DentLight (Dorset, UK), Ivoclar Vivadent Inc. (Schaan, Liechtenstein), Premium Plus UK Ltd. (Dorset, UK), Ultradent Products Inc. (Utah, UT, USA) and Woodpecker (Guangxi, China) offers different versions of their standard unit.

Optional curing modes are available in every LED-LCU, allowing dental professional control over the diverse clinical applications.

Manufacturers have assigned a wide range of trade names to the high-power display modes, such as “*High Power*”, “*Boost*”, “*Quick*”, “*Xtra Power*”, “*Turbo*”, and others, all consisting in a high radiant emittance and short-curing time light-setting of the LCU. Most of LED-LCU had radiant emittance values between 2000–3000 mW/cm^2^. The highest stated radiant emittance found was 6000 mW/cm^2^ in FlashMax^™^P3 LCU.

Automatic exposure times are typically set, however adjustable time options are available in Be Light LED^®^, LED Light Curing^®^, and LED Clear^®^. These devices allow setting the desired time in the chosen work mode (i.e., 15 to 30 s).

Seventeen LED-LCUs are able to radiate multiple wavelengths compatible with different photoinitiators, and the additional fifteen are monowave.

Regular LCU check and light emission quality is essential. Eleven LCUs have light meters built into the charging base: MiniLED^®^ Standard, MiniLED^®^ Supercharged and MiniLED^®^ Ortho 2, Bluephase^®^ Power Cure and T-LED have an incorporated radiometer while Be Light LED^®^, D-Lux+^®^, C01-D^™^, C02-D^™^, C01-S^™^, and C02-S^™^ have light intensity sensors not specified by the manufacturers. The light output can rarely be reliably measured by a commercial dental radiometer. Also, a single irradiance value cannot completely describe the output of an LCU. To overcome these limitations, calibrated spectrometer-based systems can be used. Since those devices are not generally available in dental offices, manufacturers should provide accurate information about the distribution of the radiant emittance and spectral emission across the light tip in all available settings [2].

## 4. Discussion

The use of LED technology to light-cure dental resin-based materials offers practical advantages. They are a chosen alternative to other commercially available LCUs—quartz tungsten halogen, plasma arc, and argon ion laser. LED-LCUs are compact, lightweight, portable, battery powered, energy efficient (operate for longer periods before cooling is needed), and long-lasting, which are some of the features that make LED-LCUs important for clinicians [37]. The latest high-power units incorporate the ideal features of the best LED-LCUs, and the most suggestive development is related to reducing chair-time. However, some challenges need to be addressed, in order to minimize or prevent possible adverse or side-events.

From this bibliometric systematization of in vitro and in vivo studies, it can be highlighted that the radiant exposure (the quantity of light emission over time) is more correlative to material properties and pulp temperature increase (Table 5) than to the radiant emittance parameter itself.

Hence, it remains controversially discussed whether the use of the latest LCUs with very high radiant emittance values may actually require longer exposure than the values suggested by the manufacturer to properly cure resin-based materials [6]. Some authors of the reviewed studies question the “concept of exposure reciprocity” that assumes that when applying the same radiant exposure, the degree of conversion will be the same, regardless of the irradiance level or time of exposure [36] and assume that such relationship cannot be established to resin-based dental materials when using high-power LCUs [15,16,24,25,26,27]; others, on the other hand, agree on the potential of these units in reducing irradiation time without a significant loss of material properties [10,27,28,30,31]. Some unexpected results found in the reviewed studies may be explained by the mismatch between the stated radiant emittance and the actual emitted values. Nurlatifah et al. stated that the intensity of FlashMax™ P3 was lower than that described by the manufacturers. According to the directions for use the LCU delivers 4000 mW/cm^2^ but the measured radiant emittance was only 1200 mW/cm^2^, which affected the total energy emitted [19]. Furthermore, radiant exposure and spectral emission values of LCU, as claimed by the manufacturers, may not properly have a suitable correlation with the resin-based restorative material’s higher degree of conversion and/or photo-initiators [4,5].

Moreover, the higher the radiant exposure, the more the pulp temperature increases. Despite imposed limitations such as local anesthesia that may have affected the heat dissipation, the influence of pulpal flow rate and other clinical circumstances, as different remaining dentin or enamel thickness under which resin-based materials restorations are placed, the authors concluded that a short-time exposure and high radiant emittance, contrarily to a long exposure time, might be considered neither critical nor a potential damage to the pulp vitality. According to Runnacles et al. delivering radiant exposure values >80 J/cm^2^ to the teeth might induce pulp temperature rise above the acceptable threshold of 5.5 °C; therefore, when applying high-power energy protocols and short-curing times, interval spans between each exposure are advisable to avoid consequences to pulp vitality and subsequent signs and symptoms development [32].

When assessing quality (Table 3 and Table 4), the included studies had a similar structural pattern. They reported essential data like a structured abstract, clear objectives, detailed description of methodology, statistical method applied, and the key findings compared with relevant findings from other published studies, but often failed to justify the sample size and to describe the randomization process used (if any).

As no particular LCU should be universally applied in all restorative procedures for a given time and predictably deliver optimal polymerization results, six of the identified commercialized high-power LED-LCUs (Table 2) were tested in the reviewed studies. VALO^®^ and VALO Ortho^®^ (Ultradent Products Inc., Utah, USA), FlashMax^™^P3 (CMS Dental, Copenhagen Denmark), S.P.E.C.^®^3 (Coltene Iberia S.L., Madrid, Spain), Fusion^™^ (DentLight, Dorset, UK), and Pencure 2000 (Morita Europe, Dietzenbach, Germany) were tested in vitro. Only VALO Ortho^®^ (Ultradent Products Inc., Utah, USA) performance was evaluated in vivo.

The need to increase evidence and research regarding the adverse events associated, or not, with the use of different high-power LED-LCU, from different manufacturers (Table 7) makes this subject a clear candidate for future research. Additionally, today’s dependence on technology in dentistry implies that the operator must be proficient in essential technical specifications and safe use of devices and instruments routinely applied in dental treatments. A recent survey study reported that dentist’s awareness on technical features of their LCUs, practical use and safety is unsatisfactory nowadays [38,39,40]. Likewise, there is a lack of perception on the need for monitoring and regular checking of the LED-LCU that are daily used in dental offices. Surveys of LCUs used in dental offices worldwide show that many deliver inadequate light output [37,41,42,43]. Thirteen directions for use, that must be provided by the manufactures, were not found online and were not available for analysis (Table 7). Before operation, clinicians should verify data regarding the LCU handling, safety, efficiency, technical details, and unit’s regular maintenance. Further investigations are required to improve the general knowledge level of dental professionals regarding the use and general management of high-power LED-LCUs.

## 5. Conclusions

The advantage of introducing high-power in contemporary LED-LCUs was successful in reducing resin-based dental materials’ curing time, which led to a reduction in chair-time and an increase in patient comfort during dental care. In some clinical procedures as orthodontic bracket bonding, light-curing sealants and polymerizing resin cements the use of these units is advocated and results in a similar outcome in comparison to lower irradiances, with the advantage of shorter light-curing times.

A wide range and number of high-power LCUs are available in the market, nevertheless there are still limitations to overcome. Dental professionals should be aware of the technical details and characteristics of LED-LCU units as well as the most appropriate LCU and curing-mode to the oral clinical procedure to be performed, aiming to prevent adverse events associated with their use that may influence the clinical performance of resin-based materials and/or compromise pulp tissues.

## Figures and Tables

**Table 1:** Search strategy used in each electronic database.

**Search field 1**	(“LED dental curing-lights” OR “dental curing lights “ OR “curing lights dental” OR “high-power LED” OR “high intensity LED”
AND	
**Search field 2**	(“resin-based composite resins” OR “dental resins” OR “dental materials”)

**Table 3:** Results of the assessment of in vitro studies by the use of the modified CONSORT checklist [25]. Cells marked with an asterisk “*” represent study fulfilment for the given quality assessment parameter. Cells left blank represent non-fulfilment.

	Modified CONSORT Checklist of Items for Reporting In Vitro Studies of Dental Materials														
	1	2a	2b	3	4	5	6	7	8	9	10	11	12	13	14
Park, Roulet and Heintze, 2010 [22]	*	*	*	*	*						*		*	*	
Flury et al., 2013 [10]	*	*	*	*	*						*		*	*	
Branchal et al., 2015 [31]	*	*	*	*	*						*		*	*	
Gonulol, Ozer and Tunc, 2015 [16]	*	*	*	*	*						*		*		
Haenel et al., 2015 [17]	*	*	*	*	*						*		*	*	
Watanabe et al., 2015 [33]	*	*	*	*	*						*		*		
Armellin et al., 2016 [20]	*	*	*	*	*						*		*		
Oz, Oz and Arici, 2016 [35]	*	*	*	*	*						*		*		
Peutzfeldt, Lussi and Flury, 2016 [28]	*	*	*	*	*						*		*	*	
Bilgic et al., 2017 [27]	*	*	*	*	*						*		*		
Shimokawa et al., 2017 [15]	*	*	*	*	*						*		*	*	
Udomthanaporn, Nisalak and Sawaengkit, 2017 [11]	*	*	*	*	*						*		*		
Almeida, Martins and Martins, 2018 [18]	*	*	*	*	*	*					*		*		
Daugherty et al., 2018 [14]	*	*	*	*	*						*		*		
Nurlatifah, Eriwati and Indrani, 2018 [19]	*	*	*	*	*	*					*		*		
Vinagre et al., 2019 [21]	*	*	*	*	*						*		*	*	
Sadeghyar, Watts and Schedle, 2020 [30]	*	*	*	*	*						*		*		
Rocha et al., 2021 [36]	*	*	*	*	*						*		*	*	

**Table 4:** Results of the assessment of in vivo studies by the use of the CONSORT 2010 checklist [26]. Cells marked with an asterisk “*” represent study fulfilment for the given quality assessment parameter. Cells left blank represent non-fulfilment.

	CONSORT Checklist of Items for Reporting a Randomized Trial of Dental Materials			
	Runnacles et al., 2015 [32]	Watanabe et al., 2015 [33]	Oz, Oz and Arici, 2016 [35]	Gross et al., 2020 [29]
1a				
1b	*	*	*	*
2a	*	*	*	*
2b	*	*	*	*
3a		*	*	*
3b				
4a	*	*	*	*
4b	*	*	*	*
5	*	*	*	*
6a	*	*	*	*
6b				
7a		*	*	*
7b				
8a				
8b				
9				
10				
11a				
11b				
12a	*	*	*	*
12b	*	*	*	*
13a				
13b				
14a	*			
14b				
15				
16	*	*	*	*
17a				
17b				
18				
19				
20	*	*	*	*
21	*	*	*	*
22	*	*	*	*
23				
24				
25	*			*

**Table 5:** Summary of the main adverse events described on dental resin-based materials and on patients’ oral condition.

Author, Year	Dental Resin-Based Materials				Patients’ Oral Condition
	Degree of Conversion	Diametral Tensile Strength	Elastic Modulus	Bond Strength	Temperature Changes in the Pulp
Park, Roulet and Heintze, 2010 [22]					X
Flury et al., 2013 [10]	X				
Branchal et al., 2015 [31]	X				
Gonulol, Ozer and Tunc, 2015 [16]	X				
Haenel et al., 2015 [17]	X				
Runnacles et al., 2015 [32]					X
Ward et al., 2015 [34]				X	
Watanabe et al., 2015 [33]	X				
Armellin et al., 2016 [20]					X
Oz, Oz and Arici, 2016 [35]				X	
Peutzfeldt, Lussi and Flury, 2016 [28]	X				
Bilgic et al., 2017 [27]	X		X		
Shimokawa et al., 2017 [15]	X				
Udomthanaporn, Nisalak and Sawaengkit, 2017 [11]				X	
Almeida, Martins and Martins, 2018 [18]				X	
Daugherty et al., 2018 [14]	X				
Nurlatifah, Eriwati and Indrani, 2018 [19]		X			
Vinagre et al., 2019 [21]					X
Gross et al., 2020 [29]					X
Sadeghyar, Watts and Schedle, 2020 [30]	X				
Rocha et al., 2021 [36]	X				

**Table 6:** Summary analysis of the main adverse events described on dental resin-based materials and on patients’ oral condition.

	Author, Year	Clinical Procedure	Tests Carried Out	Outcomes
Degree of conversion	Flury et al., 2013 [10]	Light curing resin cements through glass ceramics	Directly examined through FT-IR	The higher irradiance emittance resulted in similar degree of conversion compared to standard irradiance values but with the advantage of shorter curing times.
	Branchal et al., 2015 [31]	Light curing sealants	Indirectly examined through micro hardness values	Among the tested LED curing units, only VALO provided properly curing of opaque-filled and clear-filled sealants as specified by ISO 6874.
	Gonulol, Ozer and Tunc, 2015 [16]	Polymerization of resin-based tooth-colored restorative materials	Indirectly assessed through micro hardness values	VALO’s High-power mode can be recommended for clinical applications as it can shorten the time required to adequately polymerize resin-based restorative materials.
	Haenel et al., 2015 [17]	Light curing dental resins	Directly examined through FT-IR and indirectly through micro hardness values	The hardness distribution reflects the irradiance distribution of each LCU. Irradiance emittance values and exposure time do not affect the pattern of the hardness distribution, only the hardness level.
	Watanabe et al., 2015 [33]	Polymerization of dual-cured resin cement	Indirectly studied through micro hardness values	High-intensity LED units require a shorter irradiation period than halogen and standard LED curing units to obtain Knoop Hardness Numbers similar to those observed during direct irradiation.
	Peutzfeldt, Lussi and Flury, 2016 [28]	Light curing resin cements	Indirectly evaluated through micro hardness values	The irradiation protocol significantly influences micromechanical properties of dual curing resin cements due to variation of exposure time, indicating that high- irradiance light-curing has no detrimental effect on polymerization of resin cements.
	Shimokawa et al., 2017 [15]	Polymerization of resin-based composites	Directly examined through FT-IR and indirectly through micro hardness values	The irradiance level and emission spectrum affect the polymerization of resin-based composites. Equivalent or higher microhardness and degree of conversion values were achieved when standard irradiance values were used compared to high.
	Daugherty et al., 2018 [14]	Polymerization of bulk-fill composites	Directly examined through FT-IR and indirectly through depth of cure	High irradiance and short exposure time may not provide adequate depth of cure and degree of polymerization, which can lead to undesirable clinical properties on bulk-fill composites.
	Sadeghyar, Watts and Schedle, 2020 [30]	Light-curing of a bulk fill composite	Indirectly measured by micro hardness numbers	Irradiation with the standard LCU generally gave the highest KHN values for most materials however this difference was material-dependent.
	Rocha et al., 2021 [36]	Ultra-fast polymerization of bulk-fill resin-based composite RAFT-modified	Directly measured through FT-NIR and indirectly through depth of cure	No statistical difference in depth of cure and degree of conversion were found between the *Standard* and *Xtra-Power* mode, presenting both a lower depth of cure than the claimed by the manufacturer.
Elastic modulus	Bilgic et al., 2017 [27]	Curing of orthodontic adhesives	Evaluated by nano-indentation tests	Orthodontics adhesives cured with 3200 mW/cm^2^ by VALO^®^ had higher hardness and elastic modulus values than those cured with 1400 mW/cm^2^.
Diametral tensile strength	Nurlatifah, Eriwati and Indrani, 2018 [19]	Curing of packable composite resin	Calculated after loading test with a universal testing machine	The chosen irradiation protocol influences the diametral tensile strength of packable composite resin.
Bond strength	Ward et al., 2015 [34]	Brackets cured with a high-intensity LED	Determined by clinical bond failure rates	Both curing methods showed acceptable bond failure rates to be considered clinically valid.
	Oz, Oz and Arici, 2016 [35]	Metal brackets polymerized intraorally and, to extracted tooth	Evaluated by clinical bond failure rates and shear bond strength values and the adhesive remnant index	Either 10 s of light-curing with Elipar LED or 3 s with a VALO LED can safely accomplish bracket bonding.
	Udomthanaporn, Nisalak and Sawaengkit, 2017 [11]	Orthodontic brackets bonded to human premolars	Determined evaluating shear bond strength values and the adhesive remnant index	The SBS generated by VALO curing at 6 s was not significantly different from Bluephase curing at 20 s while FlashMax^™^ P3 had significantly lowest mean SBS.
	Almeida, Martins and Martins, 2018 [18]	Bracket bonding to human premolars	Determined evaluating shear bond strength values and the adhesive remnant index	Reducing exposure time from lead to significantly lower mean values of SBS, even with the use of a high-power LED-LCU. Reduction in time did not affect the amount of adhesive remnant.
Temperature changes in the pulp	Park, Roulet and Heintze, 2010 [22]	Maxillary premolar exposed to light curing units	Measured with a K-type thermocouple	Disparity in the intrapulpal peak temperature during the light-curing process and in the rate of temperature decrease in pulp after switching off the device between curing lights with different power densities was observed.
	Runnacles et al., 2015 [29]	Human premolars exposed to a light curing unit	Measured with type T thermocouple	Exposing tooth to a polywave LED-LCU develops significant increase in pulp temperature. Most exposure modes led to variations in temperature lower than the potential damage threshold temperature increase of 5.5 °C, although some teeth exposed to high radiant exposures for 60 s exhibited pulp temperature rises above 5.5 °C.
	Armellin et al., 2016 [20]	Composite restorations in first molars	Measured with with a type J thermocouple	Intrapulpal temperature increase during composite photocuring is related to the exothermic polymerization reaction, the energy from the light unit and time of exposure. Longer exposure times resulted in higher increase of pulp tissue temperature, although radiant emittance was lower.
	Vinagre et al., 2019 [21]	Immediately extracted premolars submitted to light curing procedures	Measuredwith with an Optical Fibber Bragg grating sensor	A significant pulp temperature rise was detected when intact premolars were exposed to LED-LCUs. Curing modes emitting high energy densities produced pulp temperature variations around or above 5.5 °C and might be considered clinically relevant.
	Gross et al., 2020 [29]	First premolars requiring extraction exposed to a Polywave LED LCU	Measured with a type T thermocouple	Short exposure to high radiant emittance values were not different from those when teeth were exposed to longer exposure with lower radiant emittance values, given near-similar radiant exposure values. Also, increase values similar to or even higher than the threshold temperature increase of 5.5 °C caused no noticeable histological changes in the pulp tissue.

**Table 7:** High-power LED-LCUs commercially available and technical details: manufacturer, LED-LCU, radiant emittance and exposure time of high-power curing modes, estimated wavelength range, incorporated light meter, and directions for use (DFU).

Manufacturer	LED-LCU (Ref. Number)	High-Power Curing Modes	Radiant Emittance (mW/cm^2^)	Exposure Time (s)	Wavelength Range (nm)	Light Meter Built in	DFU
**ACTEON**, France	**MINILED STANDARD^®^** (F02530)	Fast	2000	6 or 12	420–480	Yes	Available at <https://www.acteongroup.com/es/uploads/media/default/0001/01/444188825e19cff843cf686925b79f3efb247df5.pdf> (accessed on 19 February 2020)
	**MINILED SUPERCHARGED^®^** (F05217)	Fast-Cure	2000 (7.5 mm light tip) 3000 (5.5 mm light tip)	3, 4, 5 or 10	420–480	Yes	Available at <https://www.acteongroup.com/es/uploads/media/default/0001/01/45de84dd04184927973481ea27a32bd5b126e089.pdf/> (accessed on 19 February 2020)
	**MINILED ORTHO 2^®^** (F05220)	Fast Cure	3000	4, 8, 12 or 32	420–480	Yes	Available at <https://www.acteongroup.com/us/uploads/media/default/0001/01/d57b6f0c62b68188932a263f44a400fe9b383d76.pdf> (accessed on 19 February 2020)
**BA INTERNATIONAL**, UK	**ULTIMATE BASE290** (BA110200)	Power Level 3 Power Level 4	2000 3000	1, 3 or 5 1, 3 or 5	380–500	No	Available at <https://www.bainternational.com/pub/media/kuki/download/50/BASE290-IFU-FINAL.pdf> (accessed on 20 February 2020).
**BADER**, Spain	**BE LIGHT LED^®^** (09070004)	NF^1^	2000	15 to 30 (adjustable)	420–480	Yes	Available at <http://www.bader.es/gb/index.php?controller=attachment&id_attachment=69> (accessed on 21 April 2020)
	**LED LIGHT CURING^®^** (090770008)	NF^1^	2000	5 to 40 (adjustable)	420–480	No	Available at <http://www.bader.es/gb/index.php?controller=attachment&id_attachment=70> (accessed on 21 April 2020)
	**ONE LED LIGHT^®^** (09070088)	NF^1^	2300	1, 5 or 10	385–515	No	NF http://www.bader.es/gb/clinic-equipment/1243-one-led-light-bader.html?search_query=one+light&results=35 (accessed on 21 April 2020)
**CLARBEN**, Spain	**LED CLEAR^®^** (09–080)	Bright Light	2000	5 to 40 (adjustable)	420–480	No	Available at <https://clarben.com/_files/200000330-3a62b3b5bd/FT-SGC15.01%20LAMPARA%20LED%20CLEAR.pdf> (accessed on 21 April 2020)
**CMS DENTAL**, Denmark	**FLASHMAX^™^ P3** (100400)	Green Orange Red	5000 to 6000	1 or 3 1 or 3 (two activations with 0.5 s pause) 1 or 3 (repetitive cycles with 0.5 s pause)	440–480	No	NF https://www.cmsdental.com/?id=422&c=Technic%20Flash&ulang=2 (accessed on 21 April 2020)
	**FLASHMAX^™^ P3 WIDE SPECTRUM** (100403)	Green Orange Red	5000 to 6000	2 or 4 2 or 4 (two activations with 0.5 s pause) 2 or 4 (repetitive cycles with 0.5 s pause)	390–480	No	NF https://www.cmsdental.com/?id=422&c=Technic%20Flash&ulang=2 (accessed on 21 April 2020)
	**FLASHMAX^™^ P3 ORTHO** (NF)^1^	Green Orange Red	5000 to 6000	1 or 3 1 or 3 (two activations with 0.5 s pause) 1 or 3 (repetitive cycles with 0.5 s pause)	440–480	No	NF https://www.cmsdental.com/?id=422&c=Technic%20Flash&ulang=2 (accessed on 21 April 2020)
**COLTENE**, Spain	**S.P.E.C.^®^ 3** (60013942)	3K Ortho	3000 3000	1, 2 or 3 1, 2 or 3	430–490	No	Available at <https://www.coltene.com/pim/DOC/IFU/docifu40001378g-spec3-ifu-multisallaindv1.pdf> (accessed on 18 November 2019)
**DENTLIGHT**, UK	**FUSION 5^™^** (7800080)	Pulse Plasma	2000 4000	3, 5, 10, 20 or 60 3	420–490	No	Available at < http://www.dentlight.com//////////////wp-content/uploads/2019/07/FUSION5-Platform-IFU.pdf> (accessed on 22 April 2020)
	**FUSION GRAND^™^** (7830060)	Pulse Plasma	2000 4000	3, 5, 10, 20 or 60 3	385–490	No	Available at < http://www.dentlight.com//////////////wp-content/uploads/2019/07/FUSION5-Platform-IFU.pdf> (accessed on 22 April 2020) Available at < http://www.dentlight.com//////////////wp-content/uploads/2019/07/FUSION5-Platform-IFU.pdf> (accessed on 22 April 2020)
	**FUSION PLUS^™^** (7820060)	Pulse Plasma	2000 4000	3, 5, 10, 20 or 60 3	385–490	No	NF http://www.diadenteurope.com/producten/small-equipment/d-luxplus-cordless-curing-light (accessed on 22 April 2020) Available at <http://downloads.ivoclarvivadent.com/zoolu-website/media/document/46806/Bluephase+PowerCure> (accessed on 20 November 2019)
**DIADENT**, The Netherlands	**D-LUX^+®^** (4008–1110)	Max Power	2400	1, 2 or 3	385–515	Yes	Available at <http://downloads.ivoclarvivadent.com/zoolu-website/media/document/39483/Bluephase+Style+20i+-+en%2C+de%2C+fr%2C+it%2C+es%2C+pt> (accessed on 18 November 2019)
**IVOCLAR VIVADENT**, Liechtenstein	**BLUEPHASE POWER CURE^®^** (667092)	3sCure Turbo	3000 2000	3 5	385–515	Yes	NF https://www.jmoritaeurope.de/en/products/handpieces-and-instruments/curing-light/pencure/ (accessed on 22 April 2020)
	**BLUEPHASE STYLE 20i^®^** (682110)	Turbo	2000	5	385–515	No	NF https://www.myray.it/en/myray/ (accessed on 22 April 2020) NF https://www.premiumplusuk.com/product/c01-d-led-curing- (accessed on 22 April 2020)
**MORITA**, Germany	**PENCURE 2000** (NF) ^1^	High Power	2000	2 or 3	380–430	No	light-with-fibre-optic-light-guide/ (accessed on 15 November 2019) NF https://www.premiumplusuk.com/product/c02-d-led-curing- (accessed on 15 November 2019)
**MYRAY**, Italy	**T-LED** (70140020)	Standard Quick	2400 (5 mm light tip) 2200 (8 mm light tip) 3780 (5 mm light tip)	1, 2 or 3 1, 2 or 3	430–490	Yes	light-90-right-angle-direct-light-source-head/ (accessed on 15 November 2019)
**PREMIUM PLUS**, UK	**C01-DUAL RANGE^™^** (NF) ^1^	Turbo 3′ + 3′	2000 2000	4 3 (two activations with 1 s pause)	390–480	Yes	NF https://www.premiumplusuk.com/product/c01-s-led-curing-light-with-fibre-optic-light-guide/ (accessed on 15 November 2019)
	**C02-DUAL RANGE^™^** (NF) ^1^	Turbo 3′ + 3′	2500 2000	3 3 (two activations with 1 s pause)	390–480	Yes	Available at <https://www.acteongroup.com/es/uploads/media/default/0001/01/444188825e19cff843cf686925b79f3efb247df5.pdf> (accessed on 19 February 2020)
	**C01-SUPER POWER^™^** (NF)^1^	Turbo 3′ + 3′	2000 2000	4 3 (two activations with 1 s pause)	440–480	Yes	Available at <https://www.acteongroup.com/es/uploads/media/default/0001/01/45de84dd04184927973481ea27a32bd5b126e089.pdf> (accessed on 19 February 2020)
	**C02-SUPER POWER^™^** (NF) ^1^	Turbo 3′ + 3′	2500 2000	3 3 (two activations with 1s pause)	440–480	Yes	Available at <https://www.acteongroup.com/us/uploads/media/default/0001/01/d57b6f0c62b68188932a263f44a400fe9b383d76.pdf> (accessed on 19 February 2020)
**ULTRADENT**, USA	**VALO^®^** (5941)	Xtra Power	3200 (8 mm light tip)	3	395–480	No	NF https://www.premiumplusuk.com/product/c02-s-led-curing-light-90-right-angle-direct-light-source-head/ (accessed on 15 November 2019)
	**VALO GRAND^®^** (5972)	Xtra Power	3200 (12 mm light tip)	3	385–515	No	NF https://www.ultradent.com/products/procedures/restorative/class-I-II-III-IV-composite-restoration/curing-lights/valo-cordless (accessed on 23 April 2020)
	**VALO ORTHO^®^** (5942)	Xtra Power Xtra Power Q	3200 3200	1, 2 or 3 3 (five activations with 2 s pause)	395–480	No	NF http://www.glwoodpecker.com/index.php?m=content&c=index&a=show&catid=36&id=197 (accessed in 23 April 2020)
**WOODPECKER**, China	**B-CURE^®^** (NF) ^1^	Ortho	2000	3 or 5 (ten activations with 1 s pause)	385–515	No	NF http://www.glwoodpecker.com/index.php?m=content&c=index&a=show&catid=36&id=195 (accessed on 23 April 2020)
	**B-CURE PLUS^®^** (NF) ^1^	Turbo	2800 to 3000	1 or 3	385–515	No	NF http://www.glwoodpecker.com/index.php?m=content&c=index&a=show&catid=36&id=44 (accessed on 23 April 2020)
	**ILED^®^** (NF) ^1^	Turbo	2300 to 2500	1 or 3	420–480	No	NF http://www.glwoodpecker.com/index.php?m=content&c=index&a=show&catid=36&id=159 (accessed on 23 April 2020)
	**ILED PLUS^®^** (NF) ^1^	Turbo	2300 to 2500	1 or 3	385–515	No	NF http://www.glwoodpecker.com/index.php?m=content&c=index&a=show&catid=36&id=45 (accessed on 23 April 2020)
	**X-CURE^®^** (NF) ^1^	High	2300 to 2500	1, 2 or 3	385–515	No	NF https://www.premiumplusuk.com/product/c02-s-led-curing-light-90-right-angle-direct-light-source-head/ (accessed on 15 November 2019)

^1^ NF – Not found.

## Data Availability

The data presented in this study are available on request from the corresponding author.
